# Neural Field Theory of Corticothalamic Attention With Control System Analysis

**DOI:** 10.3389/fnins.2019.01240

**Published:** 2019-11-26

**Authors:** Tara Babaie-Janvier, Peter A. Robinson

**Affiliations:** ^1^School of Physics, University of Sydney, Sydney, NSW, Australia; ^2^Center of Excellence for Integrative Brain Function, University of Sydney, Sydney, NSW, Australia

**Keywords:** brain dynamics, attention, prediction, cortex, thalamus, neural field theory, control systems, data filter

## Abstract

Neural field theory is used to analyze attention by extending an existing model of the large-scale activity in the corticothalamic system to incorporate local feedbacks that modulate the gains of neural connectivity as part of the response to incoming stimuli. Treatment of both activity changes and connectivity changes as part of a generalized response enables generalized linear transfer functions of the combined response to be derived. These are then analyzed and interpreted via control theory in terms of stimulus-driven changes in system resonances that were recently shown to implement data filtering and prediction of the inputs. Using simple visual stimuli as a test case, it is shown that the gain response can implement attention by evaluating two main features of the stimuli: the magnitude and the rate of change, by increasing the weight placed on the rate of change in response to sudden changes, while reducing the contribution of stimuli value in tandem. These changes of filter parameters are shown to improve the prediction of the upcoming stimuli based on its recent time course. This outcome is analogous to controller-parameter tuning for performance enhancement in engineering control theory.

## 1. Introduction

Attention is implemented in brain by dynamically selecting specific neural activities for further evaluation, in which irrelevant sensory information is filtered out in favor of the behaviorally relevant. It has been proposed that attention can be viewed as enhancing the weight given to relevant information in a competitive system (Noudoost et al., 2010). In theory, this can be done in a number of ways, including strengthening selected signals and reducing noise (Hillyard and Anllo-Vento, 1998; Hillyard et al., 1998; Barceló et al., 2000; Noudoost et al., 2010).

Morgan et al. (1996) showed that the magnitude of steady-state visual evoke potentials (SSEVPs) recorded from the scalp of the human subjects who were cued to attend to a stimulus were significantly enlarged. Luck et al. (2000) studied the same phenomenon for single unit cells in macaque monkeys and concluded that when several simultaneous stimuli are present, a neuron's sensory response to the attended signal is significantly enhanced. This amplification is analogous to improving the signal-to-noise ratio which enhances the extraction of pertinent features of the external stimulus (Morgan et al., 1996; Hillyard and Anllo-Vento, 1998; Hillyard et al., 1998). The examples of such sensory gain control mechanisms involved in visual attention, were measured via event-related potentials (ERPs) and cerebral blood flow (positron emission tomography known as PTE, and functional magnetic resonance imaging known as fMRI) (Hillyard and Anllo-Vento, 1998; Hillyard et al., 1998; Barceló et al., 2000; Kastner and Ungerleider, 2001; Vuilleumier et al., 2001; Pinsk et al., 2004). Furthermore, top-down effects are suggested to play an important role in the processing of stimuli in corticothalamic network where they contribute to prediction of forthcoming inputs (Pessoa et al., 2003; Pinsk et al., 2004; Friston, 2005, 2010; Feldman and Friston, 2010). Engel et al. (2001) and Engel and Fries (2010) proposed that making such predictions might involve the temporal structure of both stimulus-driven and background activity, which suggested that prediction and attention are processes interconnected through synchronous oscillations. In psychology, qualitative consciousness models such Integrated Information Theory (Jerath and Beveridge, 2019) and Default Space Theory (Jerath et al., 2015) advocate a central role of corticothalamic system in dynamic and global functions with oscillations in the corticothalamic system amplifying relevant stimuli (neural facilitation of attended signals) based on the current state of internal predictions.

Despite above qualitative proposals regarding the existence of a gain control mechanisms in attention and its influence in corticothalamic neural prediction, the basic mechanisms of such modulation are poorly understood. Examples include the temporal dynamics of activity modulation (i.e., temporal corticothalamic processing), the relationship of modulation to stimulus-driven activity, the relationship of modulations to brain rhythms (i.e., how slow waves or alpha oscillations change during attention), and above all, a plausible neuronal basis by which gain calibration can be implemented. One example of this point is the Bayesian-based models of prediction and attention in the brain, motivated by machine learning applications in neuroscience along with cortical neuroanatomy and neurophysiology (Feldman and Friston, 2010; Friston, 2010), including the Kalman-filter approach used by Rao and Ballard (1999) to explain visual information processing in the brain. This method assumes linear dynamics and that Gaussian distributions describe the activities of populations of neurons, but central steps (such as complex matrix operations) have not yet been shown to be physiologically realizable in the brain's tissues.

In previous work, neural field theory (NFT) was used to determine the dynamics of corticothalamic responses to visual stimuli and it was shown that their properties are analogous to those widely used in control systems for prediction, gain tuning, and control (Babaie-Janvier and Robinson, 2018). NFT of the corticothalamic system uses the physiological properties of the brain to describe the dynamics of, and interactions between, populations of neurons in the cortex and thalamus, including feedback mechanisms (Robinson et al., 1997, 2002, 2004; Rennie et al., 2002). The equations of the NFT model are non-linear in general and they have been successfully employed to explain highly non-linear phenomena, such as epileptic seizures (Breakspear et al., 2006). However, normal brain dynamics have been shown to be able to be approximated by linear perturbations from spatially uniform steady states (Robinson et al., 1997, 2002, 2004). This approximation enabled a plethora of experimental phenomena, in cohorts of up to 2,100 subjects, to be reproduced, including evoked responses (O'Connor and Robinson, 2004; Robinson et al., 2005; Kerr et al., 2008; Van Albada et al., 2010; Roberts and Robinson, 2012; Abeysuriya et al., 2015). Neural field models of firing rate activity have been widely used to analyse the dynamics of multi-scale properties of spatio-temporal neural mechanisms (Nunez and Cutillo, 1995; Jirsa and Haken, 1996; Coombes, 2005). Certainly, linear responses account for a host of phenomena and must be thoroughly understood before proceeding to non-linear cases.

In recent work (Babaie-Janvier and Robinson, 2018) we showed that corticothalamic dynamics are dominated by alpha, beta, and slow-wave resonances, each of which can be considered as a proportional-integral-derivative (PID) filter (Ogata and Yang, 1970) that are widely used in engineering control systems to predict the future course of inputs in their relevant frequency ranges. The best prediction of the stimuli was found to be obtained by summing the filter predictions after weighting them by means of separate gain adjustments (Babaie-Janvier and Robinson, 2018). The brain, therefore, may be implementing attention through control and adjustment of input gains, which suggests that using mismatches between features of internal models and external stimuli to drive gain changes can be analogous to implementing attention in this framework. The objective of these gain adjustments, and the mechanisms by which they may be implemented in the brain is the focus of the current study.

The present work uses NFT of the corticothalamic system, based on the methods developed in (Babaie-Janvier and Robinson, 2018), to incorporate feedbacks that change synaptic gains in response to stimuli, driven by changes in pre- and/or post-synaptic activity (Koch, 1999; Rennie et al., 1999, 2002). Here we consider them as local feedbacks that directly affect the local synaptic strength. These local feedbacks are formulated in a sufficiently general way that a broad range of specific biophysical mechanisms, such as plasticity, long-term potentiation/depression, facilitation, habituation, and sensitization, can be explained by them (Rennie et al., 1999, 2002). Such biophysical effects dynamically alter the synaptic gains and their influences can be represented by dynamical equations for the evolution of the gain parameters. This yields a tractable representation of gain modulations in which the gains are dynamically adjusted as part of the system's stimulus response. We then use control theory to determine when and if gain adjustments can be interpreted as implementing attention to salient information in the stimulus by increasing gains that correspond to relevant input streams. This is done for a simple spatially unstructured visual stimulus to avoid unnecessary complexity, and explore the response of the model to impulse and step stimuli and resulting attentional changes.

This paper is organized as follows. Section 2 outlines the NFT of the corticothalamic system, the resulting transfer functions and their interpretations in terms of resonance filters. A general form of biophysically plausible mechanism for gain modulation is also briefly outlined in this section. The effect of these modulations on dispersion relations are analyzed in section 3 and interpretation of attention by gain adjustment is presented. Finally, section 4 summarizes the main findings and discusses future directions.

## 2. Materials and Methods

### 2.1. Corticothalamic Model

We first outline our original NFT model, which was developed and extensively discussed in Babaie-Janvier and Robinson (2018), and explain its key parameters. Here we briefly summarize the relevant parts of that work, including essentials of NFT and its application to a corticothalamic model that incorporates key anatomic connectivities in the cortex and the thalamus, and between them. More extensive details is found in Robinson et al. (2002), Robinson et al. (2004), and Babaie-Janvier and Robinson (2018).

**Figure 1:**
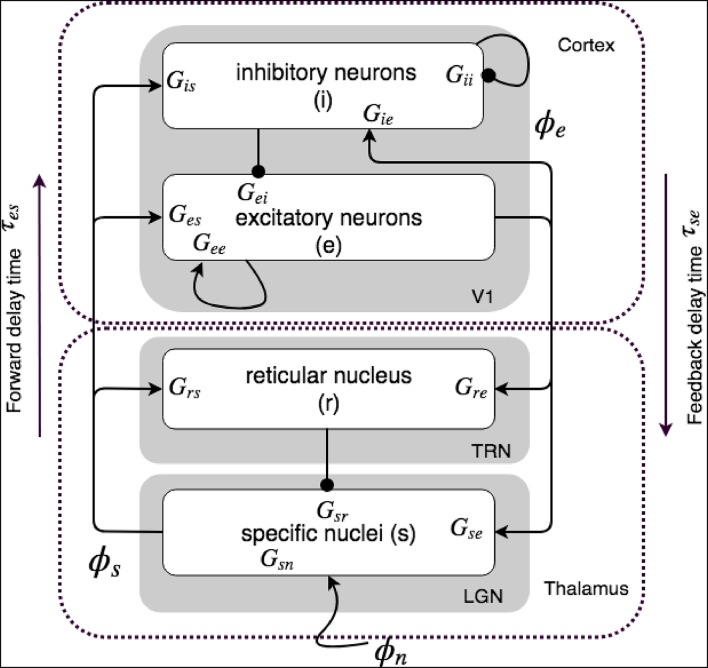
Schematics of physiologically based corticothalamic connectivities in which the arrows represent excitatory effects and the circles depict inhibitory ones. The populations are cortical excitatory (*e*) and inhibitory (*i*) neurons, the thalamic reticular nucleus (*r*), thalamic relay neurons (*s*) that project to the cortex, and non-corticothalamic neurons responsible for external inputs (*n*). The gains for impulses from neurons *b* incident on neurons of type *a* are shown as *G*_*ab*_.

Figure 1 shows our corticothalamic model which includes cortical excitatory (*e*) and inhibitory (*i*) neurons, the thalamic reticular nucleus (TRN) (*r*), thalamic relay neurons (*s*), and non-corticothalamic neurons responsible for external inputs (*n*). In this study, we consider external inputs as visuals, and the relevant relay nucleus is the lateral geniculate nucleus (LGN), where projections are to primary visual cortex (V1). The model incorporates the visual projection system with reciprocal corticothalamic feedback projections, excitatory projections to TRN from LGN-V1 feedforward axons and then V1-LGN feedback axons, and inhibitory projections from TRN onto LGN relay neurons.

NFT finds equations for evolution of dynamical variables of neural populations *a*, which are the local mean cell-body potentials *V*_*a*_, the mean rate of firing *Q*_*a*_, and the propagating axonal pulse rate fields ϕ_*a*_, by averaging over short spatial and temporal scales larger than ≈ 1*mm* (Wilson and Cowan, 1973; Freeman, 1975). Mean firing rates *Q*_*a*_ are related to mean cell body potentials *V*_*a*_(**r**, *t*) through a sigmoid function, denoted by *S*, that increases from 0 to *Q*_max_ as *V*_*a*_(**r**, *t*) increases from −∞ to ∞ and can be approximated by (Wilson and Cowan, 1973; Freeman, 1975)

(1) $$Q_{a}(r,\;t)=S[V_{a}(r,\;t)],$$

(2) $$\;=\frac{Q_{\text{max}}}{1+\text{exp }\left\{-[V_{a}(r,\;t)-\theta ]/\sigma^{\prime }\right\}},$$

where θ presents the mean threshold voltage and the standard deviation of the distribution of the threshold is given by $\sigma^{\prime }\pi /\sqrt{3}$. The response of a single neuron is thus smeared out to yield a sigmoid on the population average.

Neurotransmitter at synapses of neurons type *a* are released by arrival of signals from neurons type *b* which causes the propagation of post-synaptic voltage changes along dendrites, and soma charging, and results in the spread of temporal profile of the signals. The cell body potential at population *a*, *V*_*a*_(**r**, *t*), can thus be written as $\underset{b}{\sum }V_{ab}\left(\text{r},t\right)$, where the subscripts on *V*_*ab*_ determines the combinations of afferent neural types, and

(3) $$D_{ab}(t)V_{ab}(r,\;t)=\sum_{b}\nu_{ab}\phi_{b}(r,\;t-\tau_{ab}),$$

(4) $$D_{ab}(t)=\frac{1}{\alpha_{ab}\beta_{ab}}\frac{d^{2}}{dt^{2}}+\left(\frac{1}{\alpha_{ab}}+\frac{1}{\beta_{ab}}\right)\frac{d}{dt}+1,$$

where the temporal differential operator *D*_*ab*_ governs the temporal response of *V*_*ab*_ to afferent axonal pulse rate fields ϕ_*b*_, and β_*ab*_ and α_*ab*_ describe the rise and fall rate of the voltage at the cell body, respectively. On the right side of Equation (3), ν_*ab*_ presents the overall connection strength between two neural populations, and is given by ν_*ab*_ = *N*_*ab*_*s*_*ab*_, where *N*_*ab*_ is the average number of synapses on neurons of type *a* from neurons of type *b*, and *s*_*ab*_ is the mean time-integrated strength of soma response per incoming spike, and ϕ_*b*_(**r**, *t* − τ_*ab*_) is the mean spike arrival rate from neurons *b*, delayed by τ_*ab*_ due to discrete anatomical separations between different populations.

In NFT model, a filed ϕ_*a*_(**r**, *t*) formed by the short scale average of the neural spikes in population *a* and propagates at a velocity *v*_*a*_, obeys a damped wave equation whose source of spikes is *Q*_*a*_(**r**, *t*), with

(5) $$D_{a}(r,\;t)\phi_{a}(r,\;t)=Q_{a}(r,\;t),$$

(6) $$D_{a}(r,\;t)=\frac{1}{\gamma_{a}^{2}}\frac{\partial^{2}}{\partial t^{2}}+\frac{2}{\gamma_{a}}\frac{\partial }{\partial t}+1-r_{a}^{2}\nabla^{2},$$

where the operator *D*_*a*_(**r**, *t*) is a good approximation for the damping effect (Jirsa and Haken, 1996; Robinson et al., 1997), and γ_*a*_ is the damping rate defined by γ_*a*_ = *v*_*a*_/*r*_*a*_, in which *r*_*a*_ is the mean axon length of the population *a*, and *v*_*a*_ is the pulse velocity. In our model, only the axons of excitatory cortical neurons *r*_*e*_ are considered significantly long and cause propagation effects in Equation (6); in the other populations, we assume *r*_*a*_ ≈ 0 and therefore $D_{a}\approx 1$ which leads to ϕ_*a*_(**r**, *t*) = *Q*_*a*_(**r**, *t*) in these populations.

In our model, ν_*ie*_ = ν_*ee*_, ν_*ii*_ = ν_*ei*_, and ν_*is*_ = ν_*es*_ because in cortex, the number of synapses is closely proportional to the numbers of source and target neurons (Robinson et al., 1997; Braitenberg and Schüz, 1998), assuming the strength of synapses is determined by the source neurons. With regards to time delays, the forward delays are τ_*es*_ = τ_*is*_ ≈ 20 ms corresponding to thalamocortical propagation times and the backward delays are τ_*se*_ = τ_*re*_ ≈ 60 ms, which correspond to corticothalamic propagation times, while the remained of τ_*ab*_ are set to zero. We use a single form of *D*_*ab*_ for all the populations, i.e., the same α_*ab*_ and β_*ab*_ are assigned to every filed, which corresponds to the approximation that the mean dendritic dynamics can be described by a single pair of time constants. Note that time delays in the long-range excitatory axons in the cortex are included via Equation (6).

Table 1 lists nominal values of model parameters (Robinson et al., 2004). These values were estimated for normal adults and they have been extensively used to generate a plethora of phenomena and numerical analysis within the field, some in cohorts of up to 2,100 subjects (Rennie et al., 2002; O'Connor and Robinson, 2004; Breakspear et al., 2006; Kerr et al., 2008; Abeysuriya et al., 2015).

**Table 1:** Estimated brain parameters for normal adults in the alert, eyes-open state.

**Quantity**	**Description**	**Value**	**Unit**
*Q*_max_	Max firing rate	250	s^−1^
θ	Firing threshold	15	mV
σ′	Threshold spread	3.3	mV
γ_*e*_	Cortical damping rate	100	s^−1^
α_*ab*_	Inverse decay time	80	s^−1^
β_*ab*_	Inverse rise time	320	s^−1^
τ_*es*_	Forward delay time	20	ms
τ_*se*_	Feedback delay time	60	ms
Firing rate			
$\phi_{e}^{\left(0\right)}$	Steady-state firing rate of *e* neurons	16	s^−1^
$\phi_{s}^{\left(0\right)}$	Steady-state firing rate of *s* neurons	16	s^−1^
$\phi_{r}^{\left(0\right)}$	Steady-state firing rate of *r* neurons	16	s^−1^
$\phi_{n}^{\left(0\right)}$	Steady-state firing rate of *n* neurons	16	s^−1^
Sigmoid slope			
ρ_*e*_	For *e* neurons	4.2 ×10^3^	V^−1^ s^−1^
ρ_*s*_	For *s* neurons	4.2 ×10^3^	V^−1^ s^−1^
ρ_*r*_	For *r* neurons	6.3 ×10^3^	V^−1^ s^−1^
Synaptic gain (dimensionless)			
$G_{ee}^{\left(0\right)}$	Steady-state synaptic gain from *e* to *e*	6.8	−
$G_{se}^{\left(0\right)}$	Steady-state synaptic gain from *e* to *s*	2.5	−
$G_{ii}^{\left(0\right)}$	Steady-state synaptic gain from *i* to *i*	8.1	−
$G_{sr}^{\left(0\right)}$	Steady-state synaptic gain from *r* to *s*	−1.9	−
$G_{es}^{\left(0\right)}$	Steady-state synaptic gain from *s* to *e*	1.7	−
$G_{sn}^{\left(0\right)}$	Steady-state synaptic gain from *n* to *s*	0.8	−
$G_{ie}^{\left(0\right)}$	Steady-state synaptic gain from *e* to *i*	6.8	−
$G_{re}^{\left(0\right)}$	Steady-state synaptic gain from *e* to *r*	1.0	−
$G_{ei}^{\left(0\right)}$	Steady-state synaptic gain from *i* to *e*	−8.1	−
$G_{rs}^{\left(0\right)}$	Steady-state synaptic gain from *s* to *r*	0.19	−
$G_{is}^{\left(0\right)}$	Steady-state synaptic gain from *s* to *i*	1.7	−

*Adapted from Table I in Robinson et al. (2004), the estimated values are a self-consistent nominal set of parameters and concentrated on mean values*.

### 2.2. Steady States and Transfer Functions

The equations in the NFT of the corticothalamic model are non-linear in general, and helped study highly non-linear phenomena like seizures (Robinson et al., 2002; Breakspear et al., 2006). By setting all derivatives of the NFT equations to zero, small perturbations from spatially uniform steady-states of the system is found which have been found to correspond to normal brain states (Robinson et al., 1997, 2002, 2004; Abeysuriya et al., 2015). Stable steady-state solutions are interpreted as characterizing the baseline of normal activity, with firing rates that are in accord with experiments (Robinson et al., 2002, 2004). Linear perturbations from these neuronal steady-states represent time dependent brain activity by which numerous experimental phenomena, including evoked responses, have been reproduced (Robinson et al., 1997, 2002, 2004, 2005; O'Connor and Robinson, 2004; Kerr et al., 2008; Van Albada et al., 2010; Roberts and Robinson, 2012; Abeysuriya et al., 2015).

Low ϕ_*a*_ steady-states have been characterized with normal states of brain neuronal activity (Robinson et al., 1997) and only in considerably strong stimulation condition non-linear terms are found in the brain activities (Herrmann, 2001; Roberts and Robinson, 2012; Abeysuriya et al., 2015). We use $\phi_{a}^{\left(1\right)}$ and $V_{a}^{\left(1\right)}$ to denote linear perturbations of ϕ_*a*_ and *V*_*a*_ from their steady-state values $\phi_{a}^{\left(0\right)}$ and $V_{a}^{\left(0\right)}$. Thus, approximately,

(7) $$D_{a}(r,\;t)\phi_{a}^{\left(1\right)}(r,\;t)=\rho_{a}V_{a}^{\left(1\right)}(r,\;t),$$

where $\rho_{a}=dS\left(V_{a}^{\left(1\right)}\right)/dV_{a}^{\left(1\right)}$, evaluated at the steady state value $V_{a}^{\left(0\right)}$. The stimulus signal ϕ_*n*_ also has a steady-state component $\phi_{n}^{\left(0\right)}$ and a time-varying part $\phi_{n}^{\left(1\right)}$ that causes the response. A more detailed study of the model with respect to its parameters can be found in Robinson et al. (2004).

Applying the operator *D*_*a*_ on both sides of Equation (7), plus use of Equation (3), yield

(8) $$D_{ab}(t)D_{a}(r,\;t)\phi_{a}(r,\;t)=\sum_{b}G_{ab}\phi_{b}(r,\;t-\tau_{ab}),$$

where linear gain *G*_*ab*_ = ρ_*a*_ν_*ab*_ = ρ_*a*_*N*_*ab*_*s*_*ab*_ is the response in population of neurons *a* due to unit input from neurons *b*; i.e., the number of extra spikes out for each additional one in. The net gain of more than two populations of neurons connected serially is denoted by *G*_*abc*_ = *G*_*ab*_*G*_*bc*_.

#### 2.2.1. Transfer Functions

A linear transfer function is the ratio of the output of a linear system to its input. To obtain transfer functions one can apply either the Laplace or Fourier transform to both sides of Equation (8) for every population in the corticothalaimc system and transform it from time domain *t* to frequency domain Ω or *s*. We use single-sided Laplace transform (Ogata and Yang, 1970)

(9) $$ℒ[f(t)](s)=f(s)=\int_{0}^{\infty }f(t)e^{-st}dt,$$

where the response *e*st is parameterized by the complex frequency *s* = −*iω* = Γ − *iΩ*. We define the inverse of Laplace transformed operator in Equation (3) by

(10) $$L_{ab}(s)=D_{ab}^{-1}(s)={\left(1+\frac{s}{\alpha_{ab}}\right)}^{-1}{\left(1+\frac{s}{\beta_{ab}}\right)}^{-1},$$

where the serial filters of connected populations are *L*_*abc*_ = *L*_*ab*_*L*_*bc*_. The Laplace transform of damping operator in Equation (6) is

(11) $$D_{a}(k,\;s)={\left(1+\frac{s}{\gamma_{a}}\right)}^{2}+k^{2}r_{a}^{2},$$

where we have Fourier transformed the spatial Laplacian operator via ∇^2^ → −*k*^2^ where *k* is the wave number.

In the Laplace domain, the transfer functions to thalamus, TRN, and cortex from retina, respectively, are given by Babaie-Janvier and Robinson (2018)

(12) $$T_{sn}(k,\;s)=\frac{\phi_{s}^{\left(1\right)}(k,\;s)}{\phi_{n}^{\left(1\right)}(k,\;s)}=\frac{G_{sn}L_{sn}M_{c}(s)}{M_{c}(s)P_{t}(s)-P_{c}(s)},$$

(13) $$T_{rn}(k,\;s)=\frac{\phi_{r}^{\left(1\right)}(k,\;s)}{\phi_{n}^{\left(1\right)}(k,\;s)}=\frac{G_{sn}L_{sn}P_{r}(s)}{M_{c}(s)P_{t}(s)-P_{c}(s)},$$

(14) $$T_{en}(k,\;s)=\frac{\phi_{e}^{\left(1\right)}(k,\;s)}{\phi_{n}^{\left(1\right)}(k,\;s)}=\frac{G_{sn}L_{sn}N_{s}(s)}{M_{c}(s)P_{t}(s)-P_{c}(s)},$$

with

(15) $$M_{c}(s)=D_{ee}(1-G_{ei}L_{ii})-G_{ee}L_{ee},$$

(16) $$P_{t}(s)=1-G_{srs}L_{srs},$$

(17) $$P_{c}(s)=\left(G_{ese}L_{ese}+G_{esre}L_{esre}\right)\text{ exp }\left[-s\left(\tau_{es}+\tau_{es}\right)\right],$$

(18) Ns(s)=GesLes exp (−sτes),

(19) $$P_{r}(s)=G_{res}L_{res}\text{ exp }\left[-s(\tau_{es}+\tau_{se})\right]+G_{rs}L_{rs}M_{c}(s).$$

More details of the calculation is found in Babaie-Janvier and Robinson (2018).

Here, we calculated the transfer functions that relate corticothalamic activities to stimuli. These allow us to investigate the linear response of each population to any external input. One key feature of the transfer functions is the denominator which represents the characteristic dispersion equation of the system. Setting this equation to zero yields system's eigenvalues and mark the poles which determine the basic modes into which the system response can be decomposed. Furthermore, all transfer functions have their poles in common, as seen in Equations (12, 13), as a result of the interconnectedness of the system.

### 2.3. Corticothalamic Data Filters

The transfer functions can be decomposed into basic modes whose behaviors are shown to be associated with well-known data filters (Babaie-Janvier and Robinson, 2018). Here, we briefly outline the resulting data filters obtained for corticothalamic populations; the details are found in Babaie-Janvier and Robinson (2018). Note that only the spatially-uniform effects of perturbations (i.e., **k** = 0), were explored in the study.

If we approximate each corticothalamic transfer function *T*_*ab*_(*s*), which is a ratio of exponential polynomials of *s*, by a rational function of *s* and decompose it into partial fractions, we find

(20) $$T_{ab}(s)=\sum_{j=1}^{n}\frac{r_{j}}{s+p_{j}},$$

where, the *p*_*j*_ = Γ_*j*_ ± *iΩ*_*j*_ are all distinct poles of the system (we do not consider degenerate roots here), and the residues *r*_*j*_ = *r* ± *iΩ*_*r*_ are given by

(21) $$r_{j}=\left(s+p_{j}\right)T_{ab}\left(s\right){|}_{s=-p_{j}},$$

we estimate the reduced transfer functions $T_{ab}^{r}$ by seeking the smallest *n* that preserves the main dynamics. Babaie-Janvier and Robinson (2018) showed that a 6-pole approximation (*n* = 6) is accurate to within an root-mean-square (rms) fractional error of 0.02 over the frequency range 0–150 Hz for the parameters in Table 1. These partial fractions then are summed in pairs that dominate in slow (*f* ≲ 5 Hz), alpha (5 Hz ≲ *f* ≲ 15 Hz) and beta (15 Hz ≲ *f*) frequency regimes, respectively. We thus write (for *b* = *s, r, e*)

(22) $$T_{bn}^{r}(s)=T_{bn}^{\ell }(s)+T_{bn}^{A}+T_{bn}^{B}(s),$$

where $T_{bn}^{\ell }$ is the sum of the two real poles and represent low frequency responses while $T_{bn}^{A}$ and $T_{bn}^{B}$ are the sums over complex conjugate pairs of poles that represent oscillatory responses in the alpha and beta frequency ranges, respectively. The partial transfer function of the sum of two fractions associated with poles *p*_*j*_ and *p*_*j*+1_ either both real or conjugate pair, which we denote by $T_{ab}^{ȷ}\left(s\right)$ for ȷ=ℓ,A,B, with

(23) $$T_{ab}^{J}(s)=\left(s+\tau_{p}^{-1}\right)\left[\frac{K}{\left(s+p_{j})(s+p_{j+1}\right)}\right],$$

(24) $$\;=H_{ba}^{J}(s)I_{a}^{J}(s)$$

respectively, with τ_*p*_ = (*r*_*j*_ + *r*_*j*+1_)/(*r*_*j*_*p*_*j*+1_ + *r*_*j*+1_*p*_*j*_) and *K* = *r*_*j*_ + *r*_*j*+1_. If we write *r*_*j*_ = *r* ± *iΩ*_*r*_ for the residues at a conjugate pair of poles *p*_*j*_ = Γ ± *iΩ*_*j*_, we have

(25) $$\tau_{p}=\frac{r}{-r\Gamma +\Omega_{r}\Omega_{j}},$$

(26) K=2r.

Similarly, writing $r_{j}=r,r^{\prime }$ for the residues at real poles $p_{j}=\;\Gamma ,\Gamma^{\prime }$, we have

(27) $$\tau_{p}=-\frac{r+r^{\prime }}{r\Gamma^{\prime }+r^{\prime }\Gamma },$$

(28) $$K=r+r^{\prime }.$$

Table 2 presents the parameters calculated for three transfer functions to specific nuclei, TRN, and cortex from retina. Each slow, alpha, and beta filter captures and evaluates part of the information coming from external world, which is relevant to their frequency limit, and summing up these parallel responses results in the total response to stimulus. Each population's neural response contains an alpha resonance (7.5–12 Hz for present, adult human parameters), with peak frequencies ranging from ≈ 8.4 to ≈ 9.3 Hz, and a beta response in the beta band (12.5–30 Hz) with amplitudes significantly smaller than those of the alpha resonances (Babaie-Janvier and Robinson, 2018). Both filters present higher peak amplitudes in thalamus than cortex. Furthermore, the alpha waves persist longer than beta waves in populations *r*, *e*, and *i*, which is the direct result of the damping rates of alpha filters are approximately half of those of the beta filters. However, the results show that alpha and beta waves should last for approximately the same time in LGN structure where their damping rates are similar (Babaie-Janvier and Robinson, 2018).

**Table 2:** Characteristic parameters estimated for slow, alpha, and beta filters of corticothalamic transfer functions *T*_*an*_, for *a* = *s, r, e*, using parameters for normal healthy adults in alert, eyes-open state, adapted from Table 3 in Babaie-Janvier and Robinson (2018).

		*******T***_*****en*****_****			*******T***_*****rn*****_****			*******T***_*****sn*****_****			
**Quantity**	**Description**	**${T_{en}}^{\ell }$**	**${T_{en}}^{A}$**	**${T_{en}}^{B}$**	**${T_{rn}}^{\ell }$**	**${T_{rn}}^{A}$**	**${T_{rn}}^{B}$**	**${T_{sn}}^{\ell }$**	**${T_{sn}}^{A}$**	**${T_{sn}}^{B}$**	**Unit**
−Γ^−^	Damping rate 1st pole	9.3	14.1	26.9	10.2	13.5	27	15.3	23.8	22.3	−
−Γ^+^	Damping rate 2nd pole	17.2	14.1	26.9	14.5	13.5	27	337	23.8	22.3	−
Ω_*c*_	Characteristic frequency	0	57.4	143	0	56.2	101	0	57.9	84	s^−1^
|*K*|	Gain	1.8	3.82	1.62	7.44	4.3	8.3	52.2	48.8	4.6	−
τ_*p*_	Prediction time	16	28	6	73	7	15	18	23	2	ms

### 2.4. Synaptic Gain Modulation

The NFT model described in the previous section treated all gains that encode physiology of coupling strengths as being fixed. However, numerous biophysical processes modulate neuronal gains, dependent on current or recent level of activity, including plasticity, long-term potentiation/depression, facilitation, habituation, and sensitization. Each is considered here as a form of feedback, whereby pre- or post-synaptic neuronal activity modulates model parameters that were previously considered constant. These time-dependent effects can be included in the NFT of corticothalamic model by introducing dynamical equations for evolution of the gains; gain changes are caused by local firing rates or voltages.

We choose a general form of modulatory response that can be applied to a wide variety of specific mechanisms (Koch, 1999; Rennie et al., 1999; Robinson et al., 2002), with

(29) $$G_{ab}(r,\;t)=G_{ab}^{\left(0\right)}+G_{ab}^{\left(1\right)},$$

(30) $$\;=G_{ab}^{\left(0\right)}+g_{ab}F_{ab}(t)⊗\left[\phi_{b}(r,\;t)-\phi_{b}^{\left(0\right)}\right],$$

where $G_{ab}^{\left(0\right)}$ is the steady-state value and $G_{ab}^{\left(1\right)}$ represents the perturbation of gain caused by the feedback, where *F*_*ab*_(*t*) describes the temporal form of the modulation and the constant *g*_*ab*_ is its strength. The formulation in Equation (30) assumes that: (i) the perturbations are small enough that a linear equation is a reasonable approximation, (ii) modulation is local in space, and (iii) *g*_*ab*_ and *F*_*ab*_(*t*) do not vary with position or time. An alternative possible set of drives are the cell body potentials *V*_*a*_ (Rennie et al., 1999, 2002), but these are also linearly related to mean firing rates *Q*_*a*_ for small amplitude perturbations, so we do not consider them separately here. For the temporal form of the modulation we propose *F*_*ab*_(*t*) = η_*ab*_exp(−η_*ab*_*t*) when *t* ≥ 0 and zero otherwise to enforce causality. The rate constant η_*ba*_ characterizes the response process and η_*ab*_ > 0. This form of *F*_*ab*_(*t*) implies a simple differential equation form of Equation (30):

(31) $$\left(\frac{1}{\eta_{ab}}\frac{d}{dt}+1\right)\left[G_{ab}(r,\;t)-G_{ab}^{\left(0\right)}\right]=g_{ab}\left[\phi_{b}(r,\;t)-\phi_{b}^{\left(0\right)}\right].$$

The Laplace transform of Equation (31) is

(32) $$G_{ab}(k,\;s)=G_{ab}^{\left(0\right)}+g_{ab}F_{ab}(s)\phi_{a}^{\left(1\right)}(k,\;s),$$

where

(33) $$F(s)=\frac{1}{\tau_{ab}^{F}s+1},$$

(34) $$\tau_{ab}^{F}=\frac{1}{\eta_{ab}}.$$

The formulation developed above can be applied to different biophysical processes that change synaptic gains. For instance, Equation (32) can represent habituation if appropriate local feedback strengths *g*_*ab*_ are adapted so that a decrement in the neural activity is caused in response to stimuli sustained over several hundred milliseconds. On the other hand, if the local feedback strengths *g*_*ab*_ are tuned to increase the neuronal response due to current activity it can describe neuronal sensitisation. We further discuss the effect of feedbacks on local gains and how they modulate the neural responses in the Results section.

## 3. Results

In this section we work on deriving a general neural activity formulation which involves gain modulations via local feedbacks. The resulted activity form is then interpreted in terms of attention to more salient input. Finally, we carry out simulation using impulse and step stimuli to evaluate the corticothalamic response to various change in the information coming from external world.

### 3.1. Effect of Gain Modulation on Neural Activity

In a linear regime, the cell body potentials are related to mean firing rate through Equation (2) which gives rise to field pulses, obeying the damped wave equation in Equation (5). This results in the general firing rate equation, to first order in deviations of the variables from their steady-state values,

(35) $$\begin{array}{l} D_{ab}(t)D_{ab}(t)\left[\phi_{a}^{\left(0\right)}+\phi_{a}^{\left(1\right)}\left(r,t\right)\right]\\ \;=\sum_{b}G_{ab}(r,\;t)\left[\phi_{b}^{\left(0\right)}+\phi_{b}^{\left(1\right)}(r,\;t-\tau_{ab})\right]. \end{array}$$

The Laplace transform of Equation (35) is then

(36) $$\begin{array}{l} D_{ab}(s)\left[\phi_{a}^{\left(0\right)}+\phi_{a}^{\left(1\right)}\left(k,\;s\right)\right]=L(s)\sum_{b}\left[G_{ab}^{\left(0\right)}+g_{ab}F(s)\phi_{b}^{\left(1\right)}(k,\;s)\right]\\ \;\left[\phi_{b}^{\left(0\right)}+\phi_{b}^{\left(1\right)}(k,\;s)\text{ exp }(-s\tau_{ab})\right]. \end{array}$$

By equating orders on both left and right side of Equation (36), and omitting second order term, we find

(37) $$\phi_{a}^{\left(0\right)}=\sum_{b}G_{ab}^{\left(0\right)}\phi_{b}^{\left(0\right)},$$

(38) $$\begin{array}{l} D_{ab}(s)\phi_{a}^{\left(1\right)}\left(k,\;s\right)=L(s)\sum_{b}G_{ab}^{\left(0\right)}\phi_{b}^{\left(1\right)}(k,\;s)\text{ exp }(-s\tau_{ab})\\ \;+L(s)\sum_{b}g_{ab}\phi_{b}^{\left(0\right)}F(s)\phi_{b}^{\left(1\right)}(k,\;s)\text{ exp }(-s\tau_{ab}), \end{array}$$

(39) $$=L(s)\sum_{b}\left[G_{ab}^{\left(0\right)}+g_{ab}\phi_{b}^{\left(0\right)}F(s)\right]\phi_{b}^{\left(1\right)}(k,\;s)\text{ exp }(-s\tau_{ab}).$$

**Figure 2:**
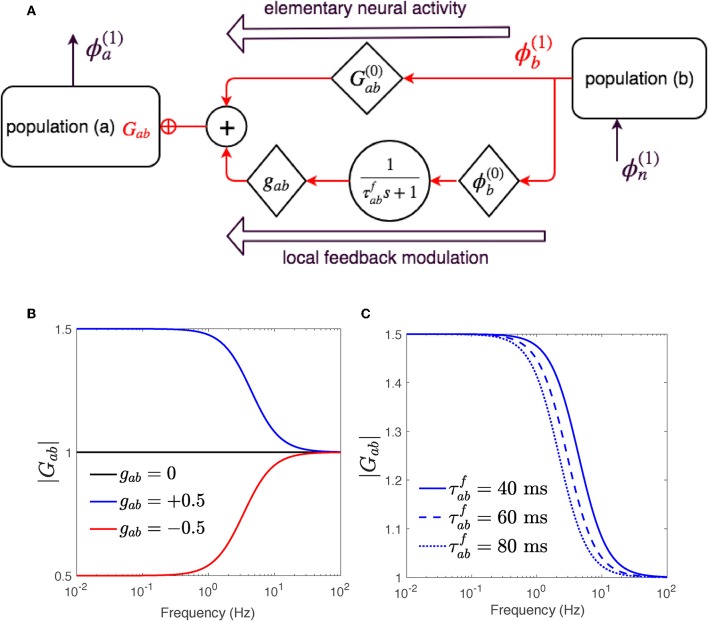
The gain *G*_*ab*_ is modulated by ϕ_*b*_ through Equation (40). **(A)** Schematic of dispersion of neural activity to population *a* from population *b*, where modulation of the neuronal gain by local feedback is given by Equation (39). **(B)** Magnitude of *G*_*ab*_ vs. frequency for various feedback strengths and $\tau_{ab}^{f}=40$ ms. **(C)** Magnitude of *G*_*ab*_ vs. frequency for various time constants $\tau_{ab}^{f}$ and *g*_*ab*_ = 0.5.

Equation (37) represents the steady-state relations. Equation (38) expresses first order responses of two types: the first term represents the part of response that would occur without change to the steady-state synaptic gains, whereas the second term represents the response due to the effect of stimulus induced gain changes on steady-state activity. Figure 2 presents a schematic of the gain modulation mechanism achieved in Equation (39).

The linear dispersion Equation (39) differs from Equation (9) only in their gain terms *G*_*ab*_. While the gains in Equation (9) comprise only fixed synaptic effect terms; i.e., $G_{ab}=G_{ab}^{\left(0\right)}$, the synaptic coefficients in the new dispersion Equation (39) contain an added modulation term $g_{ab}\phi_{b}^{\left(0\right)}F_{ab}\left(s\right)$,

(40) $$G_{ab}=G_{ab}^{\left(0\right)}+g_{ab}\phi_{b}^{\left(0\right)}F(s),$$

which results in transfer functions of corticothalamic system that represent adjustment of gains as part of the response. These new transfer functions are thus derived by substituting the term *G*_*ab*_ for *a, b* = *n, s, r, e* in Equations (18, 19) using their equivalent modulated form in Equation (40). Studying the response of the new transfer functions with two modulation terms: (i) feedback strength *g*_*ab*_, and (ii) feedback time constant η_*ab*_, requires these terms to be assigned to appropriate values. To determine a physiologically applicable range for these terms, we first focus on the process where gain is modulated by a local signal $\phi_{b}^{\left(1\right)}$, and explore how reduction or escalation of feedback strength *g*_*ab*_ and/or time evolution constant η_*ab*_ affect the gain. The applicable estimated values through these examinations can be tested by experiments, in future works.

To establish a characteristic scale of gain changes, we note that if a permanent change in $\phi_{b}^{\left(0\right)}$ occurs, the gain *G*_*ab*_ will eventually settle to a new steady-state $G_{ab}^{\left(new\right)}$, with the time evolution described by *F*_*ab*_(*s*),

(41) $$G_{ab}^{\left(new\right)}=G_{ab}(s){|}_{s\to 0},$$

(42) $$=G_{ab}^{\left(0\right)}+g_{ab}\phi_{b}^{\left(0\right)}.$$

Thus the new steady-state gain depends on the magnitude and sign of *g*_*ab*_. We define a relative coefficient of modulation, denoted by Δ, which is the magnitude of the fractional change in $G_{ab}^{\left(new\right)}$ relative to $G_{ab}^{\left(0\right)}$

(43) $$\Delta =\frac{G_{ab}^{\left(new\right)}}{G_{ab}^{\left(0\right)}}-1,$$

to investigate a variety of changes. We then choose various Δ in Equation (42) and calculate corresponding values of *g*_*ab*_ needed to produce them in the steady state. Figure 2B shows the magnitude of gains *G*_*ab*_ vs. frequency as in Equation (40) for $G_{ab}^{\left(0\right)}=1$, time constant η^−1^ = 40 ms and two positive and negative feedback strengths *g*_*ab*_ = ±0.5. As $\phi_{b}^{\left(1\right)}$ is elicited, the gain *G*_*ab*_ begins either increasing, for *g*_*ab*_ = +0.5, or decreasing for *g*_*ab*_ = −0.5 and within 40 ms reaches its new steady-state value $G_{ab}^{new}=+1.5$ or $G_{ab}^{new}=-1.5$, respectively. Figure 2C shows the magnitude of gain *G*_*ab*_ vs. frequency as in Equation (40) for $G_{ab}^{\left(0\right)}=1$, *g*_*ab*_ = 0.5, and various time evolving constant η^−1^. The smaller the η_*ab*_ the faster *G*_*ab*_ settles to $G_{ab}^{new}$.

**Figure 3:**
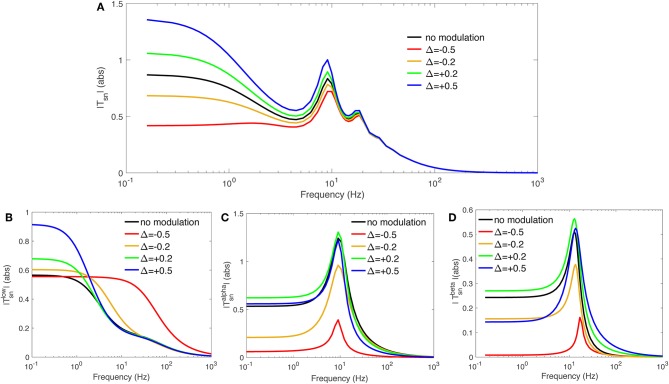
Magnitude of transfer functions *T*_*sn*_ and their derived slow, alpha, and beta-frequency filters, vs. frequency. We used varying local feedback gains *g*_*sn*_, *g*_*sr*_, and *g*_*se*_ as presented in Table 3 and the rest of parameters from Table 1. **(A)** |*T*_*sn*_|. **(B)** $\left|T_{sn}^{\ell }\right|$. **(C)** $\left|T_{sn}^{A}\right|$. **(D)** $\left|T_{sn}^{B}\right|$.

In this study we select the transfer function to specific nuclei from retina, to investigate the effect of gain modulations. The same course can be used for studying other populations. We select a mild modulation Δ = ±0.2 and a significant one Δ = ±0.5 to cover a significant range of both increased and decreased modulation effects. Table 3 presents the calculated feedback strengths *g*_*sn*_, *g*_*sr*_, and *g*_*se*_ (these are gains involved in transfer function *T*_*sn*_), that cause these amount of modulation. It should be noted that the amount of modulation, Δ, is not necessarily equal for various neuronal populations during any particular gain adjustment process, but for simplicity, we assign the same Δ in the present work. A reasonable choice for the time constant $\eta_{ab}^{-1}$ was made at 40 ms (Rennie et al., 1999; Robinson et al., 2002). Figure 3A shows the magnitude of transfer function *T*_*sn*_ vs. frequency for various values of involved local feedback gains as presented in Table 3, and using Table 1 for the rest of parameters. Figures 3B–D show slow, alpha, and beta filters calculated for these transfer functions. We use the Control System Toolbox of Matlab 2018a to carry out the calculations.

**Table 3:** Gain modulation allocated to *g*_*ab*_ for various level Δ of increased or decreased gain modulation, using parameters in Table 1 and $\eta_{ab}^{-1}=40$ ms.

**Quantity**	**Description**	**Δ** = **−0.5**			**Δ** = **−0.2**			**Δ** = **+0.2**			**Δ** = **+0.5**			**Unit**
Modulation gains														
*g*_*sn*_	Feedback strength	−0.025			−0.010			+0.010			+0.025			–
*g*_*sr*_	Feedback strength	+0.039			+0.015			−0.015			−0.039			–
*g*_*se*_	Feedback strength	−0.078			−0.031			+0.031			+0.078			–
		${T_{sn}}^{\ell }$	${T_{sn}}^{A}$	${T_{sn}}^{B}$	${T_{sn}}^{\ell }$	${T_{sn}}^{A}$	${T_{sn}}^{B}$	${T_{sn}}^{\ell }$	${T_{sn}}^{A}$	${T_{sn}}^{B}$	${T_{sn}}^{\ell }$	${T_{sn}}^{A}$	${T_{sn}}^{B}$	
Filter parameters														
−Γ^−^	Damping coefficient	137	11.1	12.1	26.1	24.2	19.18	11.5	21.4	24.8	8.7	17.7	27.2	–
−Γ^+^	Damping coefficient	259	11.1	12.1	334	24.2	19.18	338	21.4	24.8	339	17.7	27.2	–
Ω_*c*_	Cut-off frequency	0	55.7	108	0	54	87.5	0	59	83	0	58.3	83.6	s^−1^
|*K*|	Gain	11.5	363	3.9	45.9	44.8	2.21	51.9	39.1	14.1	49.9	25.7	25.6	–
τ_*p*_	Prediction time	1	363	40	9	61.3	2	19.7	16	7	18.5	12.4	23	ms

*Parameters obtained for slow, alpha, and beta filters of corticothalamic transfer functions T_an_ using the filter identification method developed in section 3*.

### 3.2. Interpreting Attention as Gain Adjustment

We have incorporated gain modulation into the corticothalamic system and showed that systems' transfer functions, and consequently its dynamics, are influenced by local feedbacks that adjust the local gains. Here we discuss how this gain adjustment can be interpreted as implementing attention in the corticothalamic system. We can write

(44) $$\phi_{a}^{\left(1\right)}=\phi_{a}^{\ell }+\phi_{a}^{A}+\phi_{a}^{B},$$

(45) $$=\left(T_{an}^{\ell }+T_{an}^{A}+T_{an}^{B}\right)\phi_{n}^{\left(1\right)}.$$

Considering the part of response associated with the filter $\phi_{a}^{J}$ for *J* of **ℓ**, A, and B, in Equation (45), and replacing the filter's transfer function from Equation (23), we have

(46) $$\phi_{a}^{J}(s)=\left(s+\tau_{p}^{-1}\right)\left[\frac{K}{\left(s+p_{j})(s+p_{j+1}\right)}\right]\phi_{n}^{\left(1\right)}(s),$$

(47) $$=K\left(s+\tau_{p}^{-1}\right)I_{a}^{J}(s)\phi_{n}^{\left(1\right)}(s),$$

(48) $$=k_{1}s\phi_{I}^{J}(s)+k_{0}\phi_{I}^{J}(s),$$

where

(49) $$\phi_{I}^{J}(s)=I^{J}(s)\phi_{n}^{\left(1\right)},$$

(50) $$k_{0}=K\tau_{p}^{-1},$$

(51) $$k_{1}=K.$$

The term *I*^*J*^(*s*) is a second-order low-pass convolution filter whose function is to allow external stimuli of a given band of frequencies to pass while attenuating or weakening all others that are not favored. In Babaie-Janvier and Robinson (2018), it was shown that *I*^**ℓ**^(*s*) is a low-pass filter while $I^{A}\left(s\right)$ and $I^{B}\left(s\right)$ are resonance filters exhibiting damped oscillations at alpha and beta rhythms, respectively.

Equation (48) shows that the convolved signal $\phi_{I}^{J}\left(s\right)$ is then modulated by two coefficients *k*_0_ and *k*_1_*s*, independently, where each generates a partial response. The inverse Laplace transform of Equation (48) yields

(52) $$\phi_{b}^{J}(t)=K\left[\frac{1}{\tau_{p}}+\frac{d}{dt}\right]\phi_{I}^{J}(t),$$

which shows the partial responses are the intensity and its rate of change, weighted by $k_{0}=K\tau_{p}^{-1}$ and *k*_1_ = *K*, respectively. The coefficients *k*_0_ and *k*_1_ determine which parts of the incoming information should be emphasized and which parts can be discarded by actively weighting the features of the stimulus, i.e., its value and rate of change. The modulation signal caused by filter gains *k*_0_ and *k*_1_, are given by

(53) $$K_{0}^{J}(t)=M\left\{k_{0}^{J}\phi_{I}^{J}(t)\right\},$$

(54) $$K_{1}^{J}(t)=M\left\{k_{1}^{J}\left[\frac{d}{dt}\phi_{I}^{J}(t)\right]\right\},$$

where M is defined as the envelop of the signals. This envelope generalizes the concept of a constant amplitude obtained for each data filter. Therefore, $K_{0}^{J}\left(t\right)$ and $K_{1}^{J}\left(t\right)$ are the modulation signals of the magnitude and the rate of change components.

**Figure 4:**
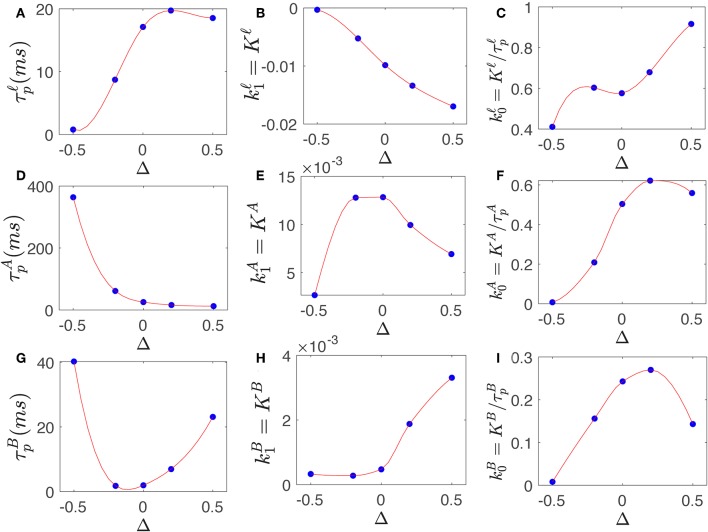
Slow, alpha, and beta filters properties change by synaptic gain changes. **(A)** Prediction time of filter $T_{sn}^{\ell }$ vs. varying Δ. **(B)** Magnitude of gain $k_{0}^{\ell }$ vs. varying Δ. **(C)** Magnitude of gain $k_{1}^{\ell }$ vs. varying Δ. **(D)** Same as **(A)** for $T_{sn}^{A}$. **(E)** Same as **(B)** for $k_{0}^{A}$. **(F)** Same as **(C)** for $k_{1}^{A}$. **(G)** Same as **(A)** for $T_{sn}^{B}$. **(H)** Same as **(B)** for $k_{0}^{B}$. **(I)** Same as **(C)** for $k_{1}^{B}$.

Figure 4 shows the filter parameters τ_*p*_ and *K* and corresponding *k*_0_ and *k*_1_ of slow, alpha, and beta filter, calculated for various level of synaptic gain changes of −0.5 ≤ Δ ≤ +0.5, using parameters in Table 3. The results show that high-frequency gains; i.e., the beta filter's *k*_0_ and *k*_1_, show inverse behavior to those of the slow and alpha filters for most Δ. For example, *k*_1_ is reduced for slow and alpha filters when Δ is increased from 0 to 0.5, whereas this weight increases for the beta filter; however, the *k*_0_ values show the opposite behavior, increasing slightly for the alpha filter and significantly for the slow filter, while decreasing for the beta filter. This confirms that by increasing the synaptic gains, the emphasis on the high frequencies is increased. The effect of these changes are explored in more detail in the next section.

The sum of slow, alpha, and beta-range responses constitute the total response to the stimulus, with

(55) $$\phi_{a}^{\left(1\right)}=\phi_{a}^{\ell }+\phi_{a}^{A}+\phi_{a}^{B},$$

(56) $$=\left(k_{0}^{\ell }I_{a}^{\ell }(s)+k_{0}^{A}I_{a}^{A}(s)+k_{0}^{B}I_{a}^{B}(s)\right)\phi_{n}^{\left(1\right)}$$

(57) $$+\left(k_{1}^{\ell }I_{a}^{\ell }(s)+k_{1}^{A}I_{a}^{A}(s)+k_{1}^{B}I_{a}^{B}(s)\right)\phi_{n}^{\left(1\right)}.$$

The inverse Laplace transform of Equations (56, 57) yields

(58) $$\phi_{a}^{\left(1\right)}(t)=\sum_{J}K^{J}\left[\frac{1}{\tau_{p}^{J}}+\frac{d}{dt}\right]\phi_{I}^{J}(t).$$

Equation (58) shows the total response is a superposition of all partial responses each scaled by a particular gain. Once a perturbation, i.e., $\phi_{n}^{\left(1\right)}$, occurs in sensory input the rate of change, i.e., $\frac{d}{dt}\phi_{I}^{J}\left(t\right)$, is significant and therefore a large part of the total response is induced by this change, whereas when the rate of change declines, i.e., $\frac{d}{dt}\phi_{I}^{J}\left(t\right)$ approaches zero, the total response is largely proportional to the magnitude of sensory input.

### 3.3. Impulse and Step Responses

Aside from the issue of how to interpret corticothalamic dynamics in terms of gain adjustment *per se*, there is the central question of how well these adjustments enable the system to pay attention to more important information in its input signals by increasing or decreasing the gains in the relevant frequency ranges. To investigate the time varying effect of local feedback in adjusting gains, we stimulate the system separately with impulse and step stimuli and track the specific nuclei's slow, alpha, and beta-frequency range responses and consequently, total firing ϕ_*sn*_. In the visual system, the impulse response is neural activity provoked by a sudden change in either the intensity (magnitude) or the rate of change (frequency) of the external stimulus that only lasts for a minimal time. It therefore corresponds to Evoked Related Potentials. A step response is neural activity stimulated by a sudden change in visual field that stays in the new level; therefore, it enables the study of steady state properties of the error signal between the external stimulus and internal model's prediction. We use varying local feedback strengths from Table 3, the system parameters from Table 1, and the Control System Toolbox Matlab 2018a to carry out the calculations.

#### 3.3.1. Impulse Response

**Figure 5:**
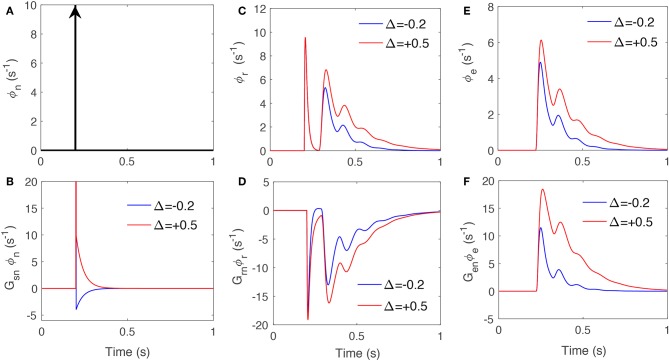
The signals that drive specific nuclei when an impulse stimulus drives the system, for varying feedback strengths presented in Table 3. **(A)** Impulse drive, ϕ_*n*_, at time 20 ms. **(B)** The response due to *G*_*sn*_ and ϕ_*n*_. **(C)** Impulse response of TRN, ϕ_*r*_. **(D)** The response due to *G*_*sr*_ and ϕ_*r*_. **(E)** The impulse response of excitatory cortex, ϕ_*e*_. **(F)** The response due to *G*_*se*_ and ϕ_*e*_.

We first drive the system with a unit impulse signal at time 200 ms. Figures 5A–F present the reticular and excitatory cortex responses when the corticothalamic system is stimulated by an impulse stimulus at time 20 ms and the signals projected in LGN through *G*_*sn*_, *G*_*sr*_, and *G*_*se*_. These show varying local feedback gains cause changes in both magnitude and latency of the evoked responses. In particular, the feedback signal from excitatory cortex shows more sensitivity to varying gains; with 50% increase in *G*_*se*_ causing the feedback signal ϕ_*e*_ to last 40 ms longer. Furthermore, the first peak is ~50% enlarged while the second peak is approximately tripled. This data supports theories in both physiological and behavioral studies (Hillyard and Anllo-Vento, 1998; Hillyard et al., 1998), which suggested that the amplitudes of stimulus-driven neural activity in sensory pathways are adjusted when tasks require attention. Gazzaley et al. (2005) provided evidence that attention not only modulates the magnitude of neural activity but also shortens the time to reach maximal response, which suggested that attention modifies the speed of neural processing.

**Figure 6:**
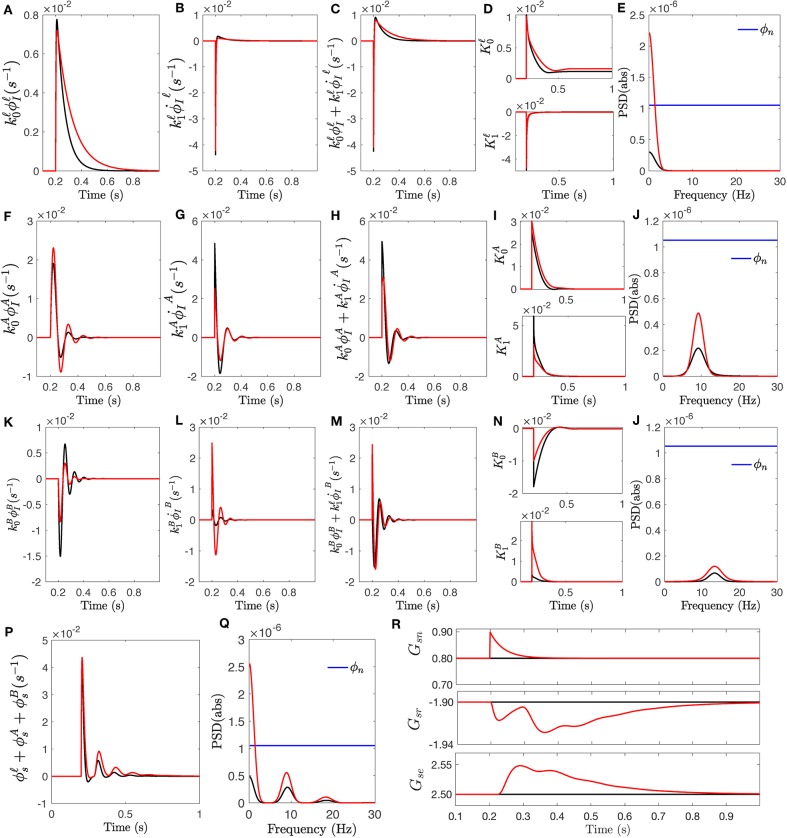
The response of transfer function *T*_*sn*_ to an impulse stimulus onsetting at *t* = 200 ms. The responses for fixed-gain transfer function (i.e., no modulation) are in black and the responses for the transfer function with modulated gains of Δ = +0, 0.5 are in red. **(A)** Slow wave response $\phi_{I}^{\ell }$ generated by convolution filter $I_{sn}^{\ell }\left(s\right)$, scaled by $k_{0}^{\ell }$. **(B)** The rate of change ${\overset{∙}{\phi }}_{I}^{\ell }$, scaled by $k_{1}^{\ell }$. **(C)** The slow filter response obtained by the sum of the two scaled signals. **(D)** Amplitude modulations vs. time for both magnitude and the rate of change of slow wave. **(E)** Power spectra of response signals obtained for $\phi_{s}^{\ell }$. **(F)** Same as **(A)** for $\phi_{I}^{A}$. **(G)** Same as **(B)** for $\phi_{I}^{A}$. **(H)** Same as **(C)** for $\phi_{I}^{A}$. **(I)** Same as **(D)** for *k*. **(J)** Same as **(E)** for $\phi_{s}^{A}$. **(K)** Same as **(A)** for $\phi_{I}^{B}$. **(L)** Same as **(B)** for $\phi_{I}^{B}$. **(M)** Same as **(C)** for $\phi_{I}^{B}$. **(N)** Same as **(D)** for *k*. **(O)** Same as **(E)** for $\phi_{s}^{B}$. **(P)** The total impulse response ϕ_*s*_. **(Q)** Power spectra of total response ϕ_*s*_. **(R)** The changes of synaptic gains involved in specific nuclei, vs. time.

In order to uncover the effect of local gain modulation, we compare the response for the fixed-gain *T*_*sn*_ with the response for the modulated-gain transfer function to a sudden change, an impulse signal. We choose the parameters for the fixed gain transfer function from Table 2 and parameters of Δ = 0.5 for the modulated transfer function from Table 3. We stimulate both systems with a unit impulse signal and in each case, we calculate the contribution of the individual slow, alpha, and beta filters and the total response ϕ_*s*_. Figure 6 shows the result in which the black curve in every plot corresponds to the original filters without gain modulation and the red curve corresponds to the modulated filters.

Once the stimulus onsets, second order convolution filters *I*^**ℓ**^, $I^{A}$, and $I^{B}$ generate specific frequency-range response; each exhibits their resonant properties. These are then scaled by $k_{0}^{\ell }$, $k_{0}^{A}$, and $k_{0}^{B}$, respectively. Figures 6A,F,D show these signals for both fixed and modulated transfer functions. The rate of change of these specific frequency-range responses are also measured by the filters and weighted by $k_{1}^{\ell }$, $k_{1}^{A}$, and $k_{1}^{B}$, shown in Figures 6B,G,L. Filter parameters plotted in Figure 4 show that the high-frequency weight $k_{0}^{B}$ significantly decreased in the modulated filter compared to the fixed filter while its weight for rate of change $k_{1}^{B}$ has increased. The slow filter wights $k_{0}^{\ell }$ and $k_{1}^{\ell }$ have shown the reverse behavior; i.e., $k_{0}^{\ell }$ has increased while $k_{1}^{\ell }$ decreased. This results in a modulated response that has less contribution from the rate of change. Similar behavior is observed for alpha filter waves, albeit less significantly. The dynamics of weights of rate of change are caused by prediction time τ_*p*_, which determine how in advance the filters estimate the future values. The prediction time of modulated high-frequency filter has increased to 23 ms from 2 ms for fixed gain filter, which shows the rapid changes are predicted sooner in advance. The alpha filter prediction time is similar in both cases. The magnitude and rate of change signals vs. time are plotted in Figures 6D,I,N.

The sum of adjusted magnitude and rate of change constitute the output of each filter, as shown in Figures 6C,H,M. As seen in these figures, once a sudden change happens (i.e., the onset of impulse function), the contribution from the rate of change component is significant at the early stage as it is seen at times between 20 and 25 ms. As the rate of change decreases, the contribution from its component also declines and the full responses of filters are mostly proportional to the value of its input. Figures 6E,J,O show the power spectra of the corresponding signals which show that modulation of both slow and alpha filters has improved the power of their corresponding rhythm bandwidth while the modulation of high-frequency (i.e., beta) filter has decreased the power of beta frequency range.

Ultimately, the total response of specific nuclei is obtained by summing the responses generated by each filter, shown in Figure 6P. The power spectra of the responses are plotted in Figure 6Q which confirms that the modulated gain filters contain more information about the stimulus. The synaptic gain changes vs. time, for those involved in specific nuclei, are plotted in Figure 6R.

#### 3.3.2. Step Response

Another aspect of stimulus-driven responses and their relation to attention, is how visual evoked potentials are related to a sudden change from an initial uniform steady state input to a new level. Hence, we drive the system by a step-function stimulus at *t* = 20 ms and again plot the specific nuclei's slow, alpha, and beta-frequency filter responses to obtain the total response ϕ_*sn*_. This enables us to study how gain modulation can modify the prediction error when a sudden change in input occurs. The Control System Toolbox of Matlab 2018a is used to carry out the calculations of time responses for both fixed and modulated gain transfer function *T*_*sn*_ using the equations and parameters from previous sections.

**Figure 7:**
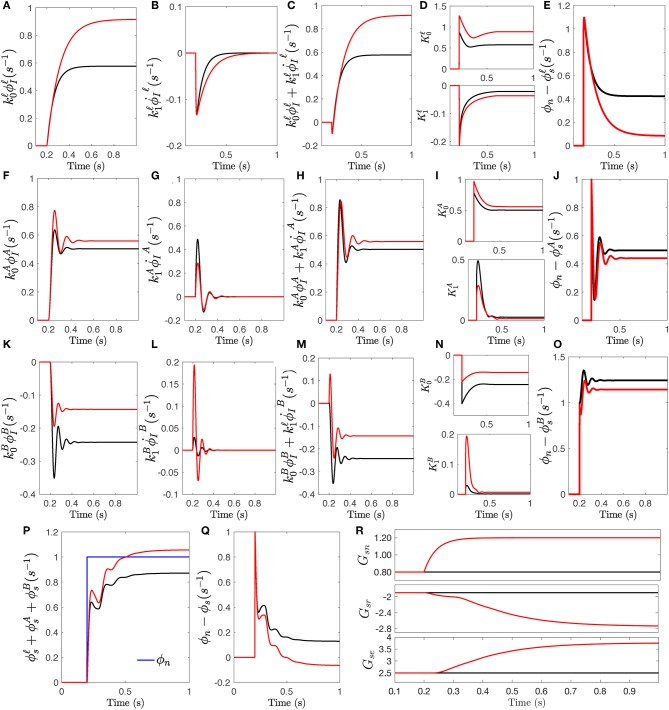
The response of transfer function *T*_*sn*_ to a step stimulus onsetting at *t* = 200 ms. The responses for fixed-gain transfer function (i.e., no modulation) are in black and the responses for the transfer function with modulated gains of Δ = +0, 0.5 are in red. **(A)** Slow wave response $\phi_{I}^{\ell }$ generated by convolution filter $I_{sn}^{\ell }\left(s\right)$, scaled by $k_{0}^{\ell }$. **(B)** The rate of change ${\overset{∙}{\phi }}_{I}^{\ell }$, scaled by $k_{1}^{\ell }$. **(C)** The slow filter response obtained by the sum of the two scaled signals. **(D)** Amplitude modulations vs. time for both magnitude and the rate of change of slow wave. **(E)** The residual signals obtained for $\phi_{s}^{\ell }$. **(F)** Same as **(A)** for $\phi_{I}^{A}$. **(G)** Same as **(B)** for $\phi_{I}^{A}$. **(H)** Same as **(C)** for $\phi_{I}^{A}$. **(I)** Same as **(D)** for *k*. **(J)** Same as **(E)** for $\phi_{s}^{A}$. **(K)** Same as **(A)** for $\phi_{I}^{B}$. **(L)** Same as **(B)** for $\phi_{I}^{B}$. **(M)** Same as **(C)** for $\phi_{I}^{B}$. **(N)** Same as **(D)** for *k*. **(O)** Same as **(E)** for $\phi_{s}^{B}$. **(P)** The total step response ϕ_*s*_. **(Q)** The residual signals obtained of total response ϕ_*s*_. **(R)** The changes of synaptic gains vs. time.

Figure 7 shows the detailed responses of modulated-gain transfer function *T*_*sn*_ and compares it with responses calculated for fixed-gain transfer function. Similar to the impulse response, each resonance filter collects a specific range of stimulus and measures the amplitude and rate of change of corresponding part of the stimulus. Figures 7A,F,K show the weighted amplitude and Figures 7B,G,L show the weighted rate of changes for each filter. The slow, alpha, and beta responses are evoked in the way similar to the impulse responses; i.e., same weights are applied to the waves. The modulation signals as the envelop of the magnitude and rate of change signals vs. time are plotted in Figures 7D,I,N. The output of filters are plotted in Figures 7C,H,M. Although both impulse and step stimuli provide similar results in transient time, because the step drive does not return to zero but remains at a new intensity, it enables the analysis of the steady-state error of the response in time domain. The errors between the stimulus and each filter's output are plotted in Figures 7E,J,O which confirms that in all three filters the error is reduced by the modulated-gain transfer function compared to the fixed-gain transfer function. The individual error reductions result in the overall error between the total response ϕ_*sn*_, plotted in Figure 7P, and stimulus being reduced, as seen in Figure 7Q. The synaptic gains changes induced by the step response are plotted in Figure 7R.

Overall, comparing the responses of the systems with and without modulation terms reveals that the key effect of modulation is to adjust the weights on features of input: its value (modulating amplification) and rate of change (modulating the latency by changing the prediction time) in such a way that new information from the external world is emphasized. These results support theories of attention that suggested that the magnitude and the processing time of stimulus-driven neural activity can be modified by attention and can provide a basis for mechanisms that explain attentional suppression and facilitation of unattended and attended inputs, respectively (Hillyard and Anllo-Vento, 1998; Hillyard et al., 1998; Gazzaley et al., 2005; Noudoost et al., 2010).

## 4. Summary and Discussion

Starting with the brain's physical characteristics we have used a neural field corticothalamic model, with modulation of neuronal gains, to evaluate the linear responses of corticothalamic neural populations to stimuli and interpret the results in terms of signal prediction and attention via control theory. In particular, control theory allows stimulus-evoked synaptic gain modulations to be interpreted as implementing attention such that salient stimulus features are emphasized, especially by increasing high-frequency gains in response to sudden stimulus changes. The main results are:

- We extended the NFT of corticothalamic system by incorporating an expression for neural gain modulation that is sufficiently general to encompass a variety of synaptic modulation mechanisms, such as long-term potentiation/depression, facilitation, habituation, and sensitization.
- Once gain modulation was incorporated as part of the system response, the system transfer functions witch were shown to differ from fixed term transfer functions only in their now-dynamic gain coefficients. These contain a fixed part, as in previous analyses, plus a gain change that occurs in response to the stimulus.
- The modulated transfer functions were then used to calculate the dominant resonant response modes that are excited by external signals. As in recent work (Babaie-Janvier and Robinson, 2018), these corresponded to slow-wave, alpha, and beta responses each of which can be interpreted as being a standard second-order PID filter, but the gain parameters of these filters can change dynamically in response to stimuli.
- Notably, it was shown that each resonance can be interpreted as a filter that responds to two features of the incoming stimulus in its resonant frequency range: the stimulus value and rate of change. These responses are then separately scaled and summed to generate the filter output, with the scalings tuned by the gain changes that occur as part of the system response. Gain increases tend to increase the weight placed on the stimulus's rate of change at the expense of its value, which we interpret as a form of attention that focuses on the feature that will better enable the future course of the stimulus to be predicted. This type of attention is fundamental, in that it involves an implicit model of stimuli as following second-order differential equations, as embodied in the transfer function's poles; understanding it is essential before progressing to more complex circumstances and stimuli.
- It was further found that weight changes of the slow and alpha filters tend to be anticorrelated with those of the beta filter, which further enhances the shift of attention from stimulus value to rate of change when there is a sudden change in the stimulus. This enables the system to respond even faster to changes in the input stimulus. These gain changes alter the strengths of the various resonances, an effect that can potentially be tested against correlations observed in evoked response experiments.
- Simulations of responses to impulse and step-function stimulus changes, with and without dynamic gain changes, verify the above points and demonstrate that attention shifts to emphasize rate of change when sudden stimulus changes occur, then back to emphasize stimulus value under static conditions. Errors in stimulus tracking and prediction were significantly reduced by dynamic gain responses relative to the fixed-gain case.

Overall, this study has demonstrated that corticothalamic responses to time-varying visual signals can be interpreted as implementing data filters that collectively span a wide frequency range, and whose parameters are dynamically adjusted, to enable estimation of incoming signals. Attention is interpreted as being due to dynamic gain changes that increase the weight attached to stimulus rate of change when sudden changes occur, and to stimulus value under static conditions. Future work will extend these insights to incorporate spatially varying stimuli, for example, and to the use of resulting stimulus predictions in decision processes.

## Data Availability Statement

The raw data supporting the conclusions of this manuscript will be made available by the authors, without undue reservation, to any qualified researcher.

## Author Contributions

TB-J and PR conceived the research and developed the theory. TB-J performed the computations. TB-J and PR verified the analytical methods. TB-J worked out the technical details, performed the numerical calculations, produced the figures, and numerical results. TB-J and PR discussed all the results. TB-J drafted the first version of the manuscript and both authors contributed to the final manuscript.

### Conflict of Interest

The authors declare that the research was conducted in the absence of any commercial or financial relationships that could be construed as a potential conflict of interest.
